# Protective effect of pentoxifylline against high-glucose-induced ferroptosis in vascular smooth muscle cells

**DOI:** 10.1530/JME-25-0086

**Published:** 2026-04-24

**Authors:** Jing Zhou, Lijing Jiao, Siyao Jin, Yiwei Ran, Xian Meng, Lu Bai, Yanru Xi, Jing Wang, Zhansheng Zhao

**Affiliations:** ^1^ Department of Endocrinology, The Second Hospital of Hebei Medical University, Shijiazhuang, Hebei, China; ^2^ Hebei Medical University, Shijiazhuang, Hebei, China

**Keywords:** pentoxifylline, high glucose, ferroptosis, vascular smooth muscle cells, diabetes

## Abstract

**Graphical abstract:**

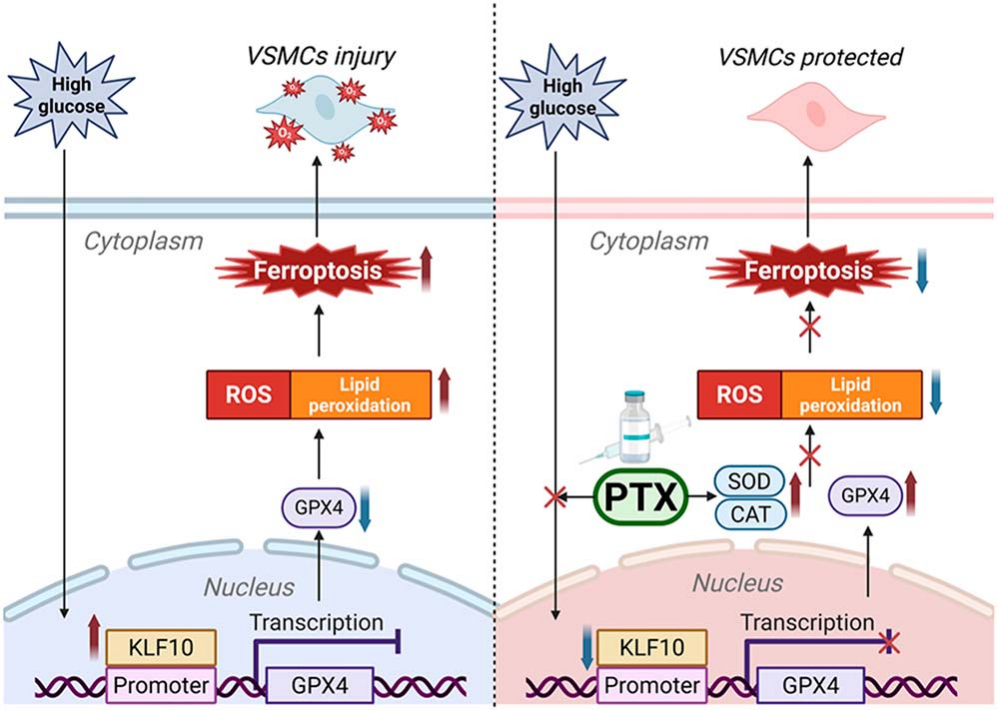

**Abstract:**

Ferroptosis has emerged as a pivotal form of regulated cell death implicated in diabetic vascular complications, yet the upstream transcriptional mechanisms governing this process remain insufficiently defined. Chronic hyperglycemia induces oxidative stress, iron overload, and vascular remodeling, but how these metabolic disturbances trigger ferroptotic signaling in vascular smooth muscle cells (VSMCs) remains unclear. In this study, we identify Krüppel-like factor 10 (KLF10) as a critical transcriptional mediator linking hyperglycemia to ferroptotic activation in VSMCs. High glucose increased KLF10 expression and enhanced its binding to the GPX4 promoter, leading to transcriptional repression of GPX4, heightened lipid peroxidation, and elevated reactive oxygen species. Pentoxifylline (PTX), a clinically used hemorheologic agent with antioxidant properties, significantly reduced ferroptosis-related lipid accumulation and partially restored GPX4 expression by suppressing KLF10 *in vitro*. In diabetic mice, PTX similarly attenuated dysregulation of the KLF10/GPX4 axis, lowered iron deposition, improved antioxidant enzyme activity, and mitigated vascular remodeling. Collectively, these findings establish the KLF10/GPX4 axis as a previously unrecognized regulator of diabetes-associated vascular ferroptosis and suggest that PTX may offer a promising therapeutic approach for limiting ferroptosis-driven vascular injury in diabetes.

## Introduction

Diabetes mellitus is a chronic metabolic disease with rapidly increasing global incidence and prevalence, characterized by persistent hyperglycemia that contributes to the development and progression of multiple complications. Vascular smooth muscle cells (VSMCs) are pivotal in maintaining vascular structure and function (1). Emerging evidence indicates that hyperglycemia renders VSMCs susceptible to ferroptosis, an iron-dependent form of regulated cell death driven by lipid peroxidation (2). Ferroptosis exacerbates vascular injury, contributing to diabetic complications, such as atherosclerosis and retinopathy (3).

A novel mode of programmed cell death discovered in recent years, ferroptosis is centrally characterized by the abnormal accumulation of iron-dependent lipid peroxides and inactivation of the glutathione peroxidase 4 (GPX4) antioxidant system (4). The role of ferroptosis in diabetes mellitus and its complications has gradually gained attention and is considered to be one of the important mechanisms of vascular injury and related complications (5). Recent studies have shown that VSMCs exhibit distinct features of iron metabolism dysfunction in response to high-glucose stimulation, including upregulation of transferrin receptor (TfR1) expression and accelerated ferritin degradation, leading to a significant increase in intracellular free iron ion levels. This iron overload state catalyzes the lipid peroxidation chain reaction via the Fenton reaction, ultimately causing irreversible damage to the cell membrane system. More importantly, the regulatory network of ferroptosis interacts in a complex manner with key signaling pathways involved in diabetic vascular complications (6). For example, inhibition of the nuclear factor E2-related factor 2 (Nrf2) pathway has been shown to exacerbate ferroptosis in diabetic nephropathy (7), and in diabetic retinopathy, ferroptosis is associated with retinal oxidative stress by inducing retinal oxidative stress, causing abnormal changes in the retinal vasculature, exacerbating retinal nerve damage, and leading to immune abnormalities (8). These findings suggest that ferroptosis may serve as an important molecular bridge between metabolic disorders and vascular injury. Ferroptosis may play an important role in the development of diabetic retinopathy, diabetic nephropathy, atherosclerosis, and other complications (9).

The transcription factor KLF10 (Krüppel-like factor 10), a member of the zinc finger transcription factor family, plays a key regulatory role in cell proliferation, differentiation, and oxidative stress response. Studies have shown that KLF10 is characteristically highly expressed in diabetic vascular tissue and is involved in vascular fibrosis through regulation of the TGF-β signaling pathway (10). Notably, KLF10 has been implicated in the antioxidant defense system (11); however, whether KLF10 is involved in the transcriptional regulation of GPX4 has not been elucidated (12). Given that GPX4 is a key regulator of ferroptosis and its expression level directly determines the cellular scavenging capacity for lipid peroxidation (13), exploring the regulatory mechanism of the KLF10–GPX4 axis in a high-glucose environment is of great value in elucidating the molecular basis of diabetic vasculopathy.

Pentoxifylline (PTX) is a synthetic methylxanthine derivative with hemorheologic, anti-inflammatory, and antioxidant properties and has received widespread attention for its unique pharmacological properties (14). Preclinical studies have shown that PTX not only has classical cardiovascular protective effects, such as improving microcirculation and inhibiting platelet aggregation, but also enhances cellular antioxidant defenses by activating adenosine A2A receptors (15). In diabetes research, PTX has been shown to attenuate high-glucose-induced mitochondrial dysfunction in renal tubular epithelial cells and to improve vascular endothelium-dependent diastolic responses in diabetic rats (16). However, studies to date have not elucidated whether PTX exerts its cytoprotective effects by regulating key pathways of ferroptosis, and in particular, its potential to regulate the KLF10/GPX4 signaling axis remains unknown.

This study aimed to investigate the protective effect of PTX against high-glucose-induced ferroptosis in VSMCs and the underlying mechanisms. In our previous study, we found that high glucose upregulated KLF10 expression in VSMCs and that KLF10 induced oxidative stress in diabetic vessels (17). Based on the available scientific background, we proposed the following hypothesis: PTX attenuates high-glucose-induced cellular ferroptosis by modulating the KLF10/GPX4 signaling axis, increasing the activities of antioxidant enzymes such as SOD and CAT, and decreasing ROS accumulation. To test this hypothesis, we established a model of high-glucose-induced ferroptosis in VSMCs, systematically investigated the transcriptional regulatory mechanism of KLF10 and its functional correlation with GPX4 expression, and elucidated for the first time the specific molecular mechanism by which PTX inhibits ferroptosis by regulating the KLF10/GPX4 axis. This study not only provides a new theoretical perspective on the pathogenesis of diabetic vascular complications but also provides an experimental basis for the development of novel therapeutic strategies based on the regulation of ferroptosis.

## Materials and methods

### Cell culture and treatment

Mouse aortic vascular smooth muscle cells (VSMCs; ATCC® CRL-2797™) were obtained from the American Type Culture Collection (ATCC, USA). Cells were maintained in Dulbecco’s modified Eagle’s medium (DMEM; Gibco, Life Technologies, USA) supplemented with 10% fetal bovine serum (Gemini Bio-Products, USA), 100 IU/mL penicillin, and 100 μg/mL streptomycin. Cultures were incubated at 37°C in a humidified atmosphere containing 5% CO_2_. When cultures reached confluence, VSMCs were detached with 0.25% trypsin–EDTA and passaged. Cells at passages 3–5 were used for all experiments. The cell suspension was adjusted to 5 × 10^6^ cells/mL, and 100 μL aliquots (5 × 10^5^ cells) were seeded into each well of 6-well plates. After allowing cells to adhere and reach ∼70% confluence, cultures were serum-starved in serum-free DMEM for 24 h to synchronize the cell cycle. To model normoglycemic and hyperglycemic conditions, low-glucose DMEM (5.5 mM D-glucose) was used as the baseline medium. High-glucose (HG) medium was prepared by supplementing low-glucose DMEM with additional D-glucose to achieve a final concentration of 25 mM. VSMCs were then exposed to either low-glucose (5.5 mM) or high-glucose (25 mM) medium for 0, 6, 12, or 24 h before harvest. Following glucose exposure, cells maintained under low- or high-glucose conditions were treated with pentoxifylline at final concentrations of 0, 5, 10, 50, 100, or 200 μM. After a 24-h treatment period, cells were collected for downstream analyses. The high-glucose concentration (25 mM) and exposure duration (0–24 h) used in this study were selected based on our previous optimization experiments. These preliminary data demonstrated that 25 mM glucose reliably induced oxidative stress, KLF10 upregulation, GPX4 suppression, and ferroptosis-associated markers in VSMCs without causing non-specific cytotoxicity. This concentration is also widely used in published studies modeling hyperglycemia-induced vascular injury.

### RNA extraction and quantitative real-time PCR

Total RNA was extracted from cells or tissues of each experimental group using the TRIzol reagent (Invitrogen, Thermo Fisher Scientific, USA) according to the manufacturer’s instructions. RNA concentration and purity were assessed by measuring the A260/A280 ratio with a NanoDrop ND-1000 spectrophotometer (Thermo Fisher Scientific, USA). For cDNA synthesis, 1–3 μg of total RNA was reverse-transcribed in a 20 μL reaction using the M-MLV First Strand cDNA Synthesis System for qRT-PCR (Invitrogen, USA) following the manufacturer’s protocol. Quantitative real-time PCR was performed on an ABI 7,500 Fast Real-Time PCR System (Applied Biosystems, USA) using the Platinum SYBR Green qPCR SuperMix-UDG with ROX (Invitrogen, USA). All reactions were run in triplicate, and relative gene expression levels were calculated using the 2^–ΔΔCt^ method with β-actin (or GAPDH) as the endogenous control.

### Western blot

Total protein was extracted from each experimental group’s cells (or tissue samples) using RIPA lysis buffer containing protease and phosphatase inhibitors. Protein concentration was determined by the bicinchoninic acid (BCA) assay. Equal amounts of protein (20–30 μg per lane) were resolved by SDS-polyacrylamide gel electrophoresis and then transferred onto PVDF membranes using a semi-dry transfer apparatus. After transfer, membranes were blocked in Tris-buffered saline with 0.05% Tween-20 (TBS-T; 10 mM Tris–HCl, pH 8.0; 150 mM NaCl; 0.05% Tween-20) containing 5% non-fat dry milk at room temperature for 2 h. Membranes were then incubated overnight at 4°C with the following primary antibodies diluted in blocking buffer: rabbit anti-KLF10 (1:1,000; ab184182, Abcam), rabbit anti-GPX4 (1:1,000; T56959, Abmart), and mouse anti-β-actin (1:10,000; 66009-1-lg, Proteintech, USA). β-actin served as the loading control. After three washes in TBS-T (5 min each), membranes were incubated with HRP-conjugated secondary antibodies (Cell Signaling Technology, USA) at room temperature for 1 h. Following another series of washes, antibody-bound protein bands were visualized using enhanced chemiluminescence (ECL) reagents (Millipore, Germany). Band intensities were quantified by densitometry using ImageJ software.

### Cell transfection

When VSMCs reached 60–70% confluence, they were transfected using Lipofectamine™ 2000 (Invitrogen, USA) according to the manufacturer’s instructions. Cells received one of the following treatments: 50 nM small interfering RNA targeting KLF10 (si-KLF10) or non-targeting control siRNA, or 2 μg per well of either the KLF10 overexpression plasmid (pcDNA3.1–KLF10) or empty pcDNA3.1 vector. The pcDNA3.1–KLF10 construct – based on the human KLF10 coding sequence – was synthesized by Shanghai Gima Biotechnology Co., Ltd (China). For each well of a six-well plate, Lipofectamine™ 2000 and the nucleic acid reagent were each diluted in 100 μL Opti-MEM® I (Thermo Fisher Scientific), gently mixed, and incubated at room temperature for 20 min to allow complex formation. Transfection complexes were then added dropwise to the cells, which were incubated for 6 h at 37°C before replacing the medium with complete growth medium. Transfection efficiency was assessed 48 h later by qRT-PCR and/or Western blotting.

### Dual-luciferase reporter assay

Cells were seeded in 24-well plates and grown to approximately 90% confluence. For each well, 0.5 μg of the pGL3-GPX4-promoter reporter plasmid, 0.05 μg of the pRL-TK Renilla control plasmid, and 0.5 μg of the KLF10 expression plasmid (or empty vector control) were co-transfected using Lipofectamine™ 2000 (Invitrogen) according to the manufacturer’s instructions. Transfections were performed in triplicate for each treatment group. Twenty-four hours after transfection, Firefly and Renilla luciferase activities were measured sequentially using the Dual-Glo® Luciferase Assay System (Promega, USA) on a luminometer capable of dual-reporter detection. Promoter activity was calculated as the ratio of Firefly to Renilla luminescence for each well.

### Chromatin immunoprecipitation (ChIP)

VSMCs (∼1 × 10^7^ cells per reaction) were cross-linked with 1% formaldehyde for 10 min at room temperature, followed by quenching with 0.125 M glycine for 5 min. Cells were washed with cold PBS and lysed in ChIP lysis buffer. Chromatin was sheared using a Bioruptor Pico sonicator (30 s ‘on’/30 s ‘off’ cycles for 10 min) to obtain DNA fragments. Equal aliquots of chromatin were incubated overnight at 4°C with rotation using KLF10 antibody (Santa Cruz Biotechnology, USA, sc-130408; dilution 1:100) or normal rabbit IgG as a negative control. DNA–protein complexes were captured using protein A/G magnetic beads (Thermo Fisher Scientific), washed sequentially with low-salt, high-salt, and LiCl wash buffers, and eluted from beads. Cross-links were reversed at 65°C for 6 h, followed by RNase A treatment at 37°C for 30 min and proteinase K digestion at 55°C for 1 h. Purified DNA was obtained using a spin-column DNA purification kit (Qiagen, Netherlands).

Quantitative PCR was performed for GPX4 promoter regions using the following primer pair targeting the predicted KLF10-binding site: forward: 5′-CTG​GTG​CTG​TTG​GTG​GTG​TT-3′; reverse: 5′-GGA​GGA​CAG​GCA​GTA​CTT​CC-3′.

Data were analyzed using the percentage input method with normalization to IgG control. ChIP experiments were conducted in triplicate biological replicates.

### MTT/CCK-8 assay

Cell viability and proliferation were evaluated using MTT and CCK-8 assays. VSMCs in the exponential growth phase were seeded into 96-well plates and subjected to the indicated treatments. For the MTT assay, 10 μL MTT solution (Sigma-Aldrich, Germany, M2128) was added for 4 h at 37°C, after which formazan crystals were solubilized in DMSO and absorbance was measured at 570 nm. For the CCK-8 assay, 10 μL CCK-8 reagent (Yeasen, 40203ES60) was added for 2 h at 37°C and absorbance was recorded at 450 nm. Measurements were obtained using a Multiskan FC microplate reader (Thermo Fisher Scientific). All conditions were performed in triplicate, and viability was calculated relative to untreated controls.

### ROS assay (DCFH-DA fluorescent probe assay)

Intracellular reactive oxygen species (ROS) levels were quantified using the DCFH-DA ROS Detection Kit (Beyotime, China) following the manufacturer’s instructions. Briefly, logarithmically growing VSMCs were seeded into 96-well plates and allocated into four groups: control, high-glucose (25 mM), control + pentoxifylline, and high-glucose + pentoxifylline. After treatment, cells were incubated with 10 μM DCFH-DA probe in serum-free medium at 37°C for 30–60 min in the dark. Wells were then washed twice with PBS to remove excess probe. Fluorescence intensity was measured at an excitation wavelength of 485 nm and an emission wavelength of 530 nm using a fluorescence microplate reader or analyzed by fluorescence microscopy/flow cytometry. ROS levels were calculated from fluorescence readings and expressed relative to the control group. All assays were performed in triplicate.

### Prussian Blue staining

Intracellular ferric iron accumulation in VSMCs was assessed using an enhanced Prussian Blue iron staining kit (Prussian Blue Iron Stain Kit, Ferric Iron, Enhanced; Cat. No. G1428, Solarbio, China). Cells grown on glass coverslips were washed with PBS, fixed with 4% paraformaldehyde for 15 min at room temperature, and rinsed thoroughly. The Perls working solution was freshly prepared by mixing solution A1 and solution A2 (1:1), and cells were incubated with this mixture at 37°C for 20 min. After brief washes with distilled water, chromogenic development was performed by incubating cells with reagent B at 37°C for 10–20 min. To further enhance signal intensity, the enhanced working solution – freshly prepared by mixing reagent C1, reagent C2, and 1× PBS (1:1:18) – was applied for an additional 10–20 min at 37°C, with monitoring to avoid overstaining. Cells were then rinsed with PBS and distilled water, air-dried, and mounted with neutral resin. Ferric iron deposits appeared as yellow-brown to dark brown granules under light microscopy. All staining procedures were performed in triplicate for reproducibility.

### Lipid peroxidation assay (MDA assay)

Lipid peroxidation was quantified by measuring malondialdehyde (MDA) levels using an MDA assay kit (Solarbio, China) following the manufacturer’s instructions. Cell or tissue lysates were prepared and reacted with thiobarbituric acid (TBA), and absorbance of the MDA–TBA adduct was measured at 532 nm to calculate MDA concentration based on a standard curve. All measurements were conducted in triplicate.

### Antioxidant enzyme activity assays (SOD and CAT)

The activities of superoxide dismutase (SOD) and catalase (CAT) were assessed using commercial assay kits (Solarbio, China) according to the manufacturer’s protocols. Cells were lysed and clarified by centrifugation, and enzyme activities were determined in the resulting supernatants. SOD activity was quantified using the nitroblue tetrazolium (NBT) inhibition method, whereas CAT activity was determined based on the rate of H_2_O_2_ decomposition. All measurements were performed in triplicate and normalized to total protein content (BCA assay).

### Animal experiments

Male C57BL/6J and db/db mice (6–8 weeks old) were obtained from Jiangsu Jicui Yikang Bio-Technology Co., Ltd, and housed in the SPF barrier facility of Hebei Medical University (with ethical approval), at 25°C, 35% humidity, and a 12 h light:12 h darkness cycle with access to water *ad libitum*. Wild-type mice (random blood glucose <7.0 mmol/L) received standard chow, whereas db/db mice (random blood glucose >11.1 mmol/L) were fed a high-fat diet for 16 weeks to induce diabetic vascular injury; thereafter, each genotype was randomized to receive daily intraperitoneal injections of pentoxifylline (60 mg/kg) or equal volumes of 0.9% NaCl for an additional 8 weeks (*n* = 6 per group), for a total study duration of 24 weeks. Body weight and blood glucose were monitored at predefined time points throughout the study, and the corresponding metabolic data are provided in Supplementary Fig. S1 (see section on Supplementary materials given at the end of the article). Twenty-four hours after the final dose, mice were anesthetized and euthanized by protocols approved by the Institutional Animal Care and Use Committee of Hebei Medical University, conforming to the Guide for the Care and Use of Laboratory Animals (NIH Publication No. 85-23, revised 1996); the thoracic aorta and other tissues were rapidly excised, rinsed in ice-cold PBS, and either fixed in 4% paraformaldehyde for histological analysis or snap-frozen in liquid nitrogen for molecular studies.

### Oil Red O staining

Mouse aortic tissues were harvested and immediately fixed in 4% paraformaldehyde for 24 h at room temperature. Following fixation, the tissues were rinsed with distilled water and then immersed in 60% isopropanol for 5 min to facilitate subsequent staining. An Oil Red O working solution (O8010, Solarbio, China) was freshly prepared as follows: 0.5 g Oil Red O was dissolved in 100 mL isopropanol, diluted with distilled water at a ratio of 3:2, filtered through a 0.45 μm filter, and pre-warmed to 60°C. The aortic tissues were incubated in the warmed Oil Red O solution for 30 min at 37°C in a light-protected environment to stain neutral lipids. Post-staining, the tissues were briefly rinsed with 60% isopropanol to remove excess dye and then washed with distilled water. The stained tissues were mounted on glass slides using an aqueous mounting medium and examined under a light microscope. Lipid-rich areas appeared as red-stained regions, indicating lipid accumulation.

### Hematoxylin and eosin (H&E) staining

Mouse aortic tissues were fixed in 4% paraformaldehyde for 24 h at room temperature, dehydrated through graded ethanol, cleared in xylene, and embedded in paraffin. Paraffin blocks were sectioned at 5 μm thickness, and sections were deparaffinized in xylene, rehydrated through 100, 95, 80, and 70% ethanol, and then rinsed in distilled water. Slides were stained with hematoxylin for 5 min, differentiated in 0.3% acid alcohol, blued in 0.1% ammonia water, and counterstained with eosin for 30 s. After dehydration and clearing, slides were mounted in neutral resin and examined under light microscopy for histopathological analysis.

### Immunofluorescence staining

Sections were deparaffinized in xylene and rehydrated through graded ethanol solutions. Antigen retrieval was conducted by boiling the sections in sodium citrate buffer (pH 6.0) for 15 min, followed by cooling to room temperature and washing with phosphate-buffered saline (PBS). To block non-specific binding, sections were incubated with 10% normal goat serum (KPL, USA) at room temperature for 1 h. Subsequently, sections were incubated overnight at 4°C with rabbit anti-GPX4 primary antibody (ab125066, Abcam, UK). After washing with PBS, sections were incubated with a rhodamine-conjugated goat anti-rabbit IgG secondary antibody (031506, KPL, USA) at room temperature for 90 min in the dark. Nuclei were counterstained with DAPI (157574, MB Biomedical, USA), and sections were mounted using an anti-fade mounting medium. Fluorescence images were captured using a confocal laser scanning microscope (DM6000 CFS, Leica, Germany).

### Statistical analysis

Statistical analyses were performed using GraphPad Prism, version 8.0 (GraphPad Software, USA). Data are presented as mean ± standard error of the mean (SEM) from at least three independent experiments. Comparisons between two groups were conducted using an unpaired Student’s *t*-test, whereas multiple group comparisons were evaluated by one-way analysis of variance (ANOVA) followed by Tukey’s post hoc test, with *P* < 0.05 considered statistically significant. All *in vitro* experiments were conducted with *n* ≥ 3 independent biological replicates, which provides adequate statistical power given the large and consistent effect sizes observed in our preliminary studies. *In vivo* experiments used *n* = 6 mice per group, based on variability assessments from pilot work and aligned with the 3R principle to minimize animal use while ensuring sufficient power to detect biologically meaningful differences.

## Results

### Pentoxifylline attenuates high-glucose-induced ferroptosis in VSMCs via the KLF10/GPX4 pathway: *in vitro* validation

**Figure 1:**
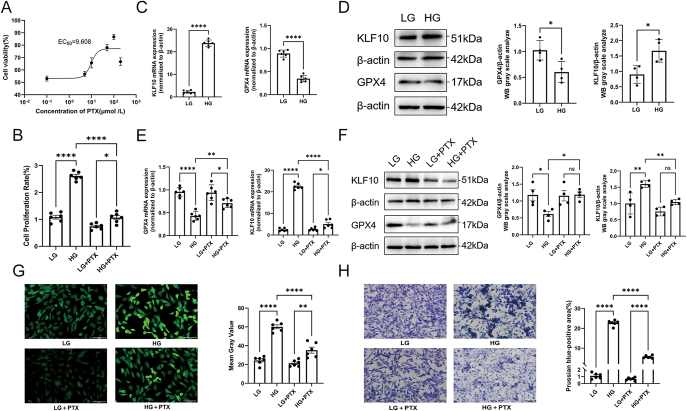
PTX mitigates high-glucose-induced ferroptotic injury in VSMCs. (A) Dose–response curve of PTX on VSMC viability assessed by the MTT assay. (B) Cell proliferation of VSMCs was assessed using the CCK-8 assay under high-glucose conditions and following PTX treatment. (C, D, E, F) Analysis of KLF10 and GPX4 expression in VSMCs exposed to high-glucose conditions and treated with different concentrations of PTX. mRNA levels were measured by RT-qPCR, and protein abundance was assessed by western blotting. (G) Intracellular ROS levels measured by DCFH-DA fluorescence staining. Scale bar = 100 μm. (H) Prussian Blue staining of intracellular iron deposition. Scale bar = 100 μm. Data are shown as mean ± SEM (*n* ≥ 3). One-way ANOVA with Tukey’s post hoc test: **P < 0.01, ****P < 0.0001. A full colour version of this figure is available at https://doi.org/10.1530/JME-25-0086.

To investigate whether high glucose induces ferroptosis and whether PTX can reverse this process, cellular phenotype and ferroptosis-associated molecular markers were assessed (Fig. 1A, B, C, D, E, F, G, H). MTT and CCK-8 analyses demonstrated that high glucose significantly enhanced VSMC proliferation. PTX treatment reduced this proliferative response in a dose-dependent manner, restoring cell viability toward that of the control group (Fig. 1A and B). At the transcriptional level, high glucose markedly upregulated KLF10 expression while suppressing GPX4 mRNA abundance. PTX progressively restored these alterations toward basal expression patterns. Consistently, western blot analysis demonstrated increased KLF10 protein levels and decreased GPX4 expression in high-glucose-stimulated VSMCs; PTX dose-dependently reversed both trends (Fig. 1C, D, E, F). In addition to transcriptional and protein effects, functional ferroptosis-associated indices were altered. High glucose markedly increased intracellular reactive oxygen species (ROS) levels, as visualized by DCFH-DA fluorescent staining, while PTX treatment reduced ROS accumulation (Fig. 1G). High-glucose exposure also induced substantial intracellular iron deposition, demonstrated by Prussian Blue staining, and PTX significantly attenuated iron accumulation (Fig. 1H). Collectively, these findings indicate that PTX mitigates high-glucose-induced ferroptotic injury in VSMCs at transcriptional, protein, and functional levels.

### Mechanistic insights into KLF10-mediated transcriptional repression of GPX4 in regulating ferroptosis: *in vitro* analysis

**Figure 2:**
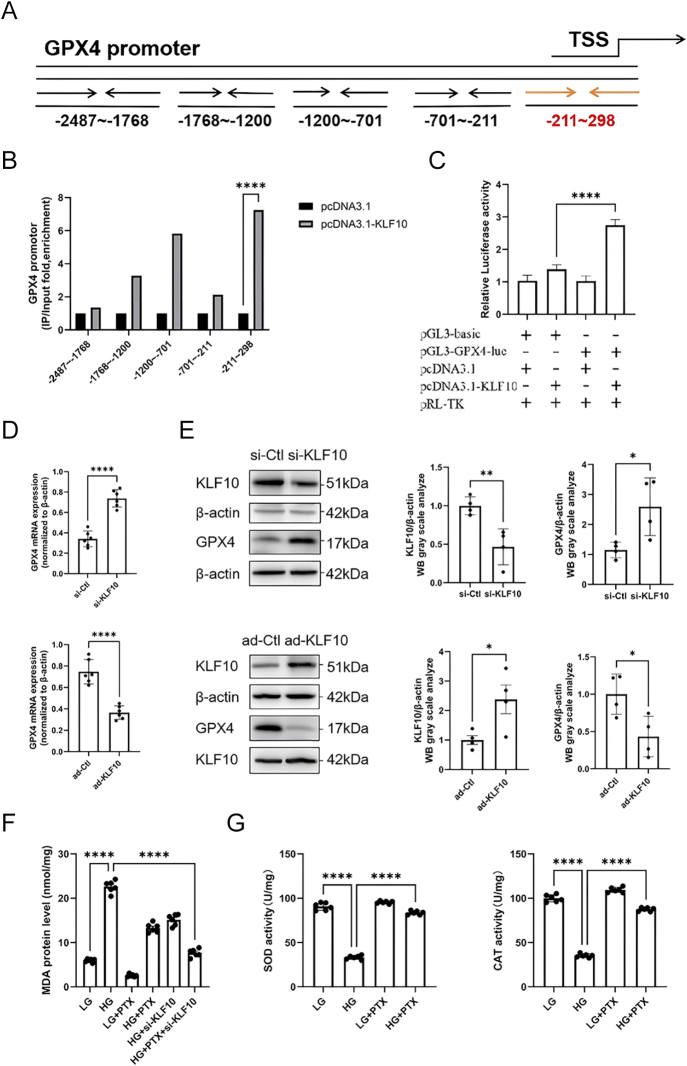
KLF10 represses GPX4 and modulates ferroptosis-associated oxidative indices. (A) Schematic overview of the GPX4 promoter structure with predicted KLF10-binding sites. (B) ChIP-qPCR showing increased KLF10 occupancy on GPX4 promoter regions following HG exposure. (C) Luciferase reporter assays demonstrating KLF10-mediated suppression of GPX4 promoter activity. (D and E) Effects of KLF10 overexpression or knockdown on GPX4 mRNA and protein abundance. VSMCs were transfected with KLF10 expression plasmid or KLF10-targeting siRNA, and GPX4 levels were assessed by RT-qPCR and western blotting. (F) Malondialdehyde (MDA) levels in VSMCs were measured using a commercial lipid peroxidation assay kit. (G) Superoxide dismutase (SOD) and catalase (CAT) activities in VSMCs were assessed using enzyme activity assay kits according to the manufacturer’s instructions. Data are shown as mean ± SEM (*n* ≥ 3). One-way ANOVA with Tukey’s post hoc test: **P < 0.01, ****P < 0.0001. A full colour version of this figure is available at https://doi.org/10.1530/JME-25-0086.

To dissect how KLF10 drives ferroptosis via transcriptional control of GPX4 – and how pentoxifylline (PTX) can counteract this – we performed the following *in vitro* studies in VSMCs under high-glucose (HG) conditions (Fig. 2A, B, C, D, E, F, G). Chromatin immunoprecipitation coupled with qPCR (ChIP-qPCR) demonstrated that KLF10 binds directly to the GPX4 promoter, and this promoter occupancy is significantly increased upon HG treatment, indicating that KLF10 acts as a direct transcriptional repressor of GPX4; motif analysis and peak mapping localized the putative KLF10-binding region to approximately −211 to −298 bp upstream of the GPX4 transcription start site (Fig. 2A, B, C). Forced overexpression of KLF10 in VSMCs markedly decreased GPX4 mRNA and protein levels, whereas siRNA-mediated knockdown of KLF10 restored GPX4 expression, confirming that KLF10 negatively regulates GPX4 at both the transcriptional and translational levels (Fig. 2D and E). HG treatment led to a significant rise in malondialdehyde (MDA), a marker of lipid peroxidation; PTX co-treatment dose-dependently suppressed MDA accumulation, indicating effective inhibition of lipid peroxidation and attenuation of ferroptotic stress (Fig. 2F). To further characterize the functional relevance of this regulatory pathway, biochemical markers associated with ferroptotic oxidative stress were evaluated. Lipid peroxidation was assessed by quantification of malondialdehyde (MDA), and antioxidant capacity was evaluated by measuring superoxide dismutase (SOD) and catalase (CAT) activities. High glucose increased MDA and suppressed SOD and CAT activity, and PTX partially restored these enzymatic and biochemical parameters (Fig. 2G). These data demonstrate that KLF10 exerts transcriptional repression on GPX4, thereby contributing to ferroptosis-associated metabolic alterations under hyperglycemic conditions.

### Pentoxifylline alleviates VSMC ferroptosis via the KLF10/GPX4 axis in diabetic mouse models: *in vivo* validation

**Figure 3:**
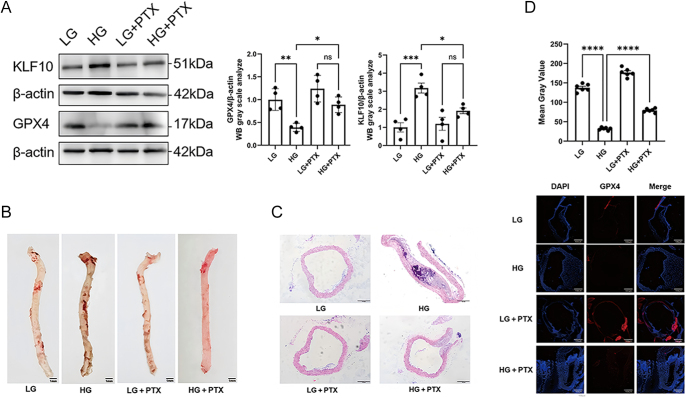
PTX alleviates ferroptosis-associated vascular remodeling in diabetic mice. (A) Western blot analysis of aortic KLF10 and GPX4 expression in diabetic and PTX-treated mice. (B) Oil Red O staining of aortic lipid accumulation. (C) Hematoxylin–eosin staining of aortic wall thickness and morphology. (D) Immunofluorescence showing GPX4 expression in aortic sections. Scale bar = 100 μm. Data are shown as mean ± SEM (*n* = 6). Statistical analysis: one-way ANOVA with Tukey’s post hoc test: **P < 0.01, ****P < 0.0001. A full colour version of this figure is available at https://doi.org/10.1530/JME-25-0086.

To evaluate the *in vivo* relevance of these findings, aortic tissues from diabetic mice with or without PTX treatment were examined (Fig. 3A, B, C, D). Western blot analysis showed that diabetes significantly increased aortic KLF10 expression while reducing GPX4 protein abundance; PTX treatment restored both markers toward control levels (Fig. 3A). Histological staining revealed structural abnormalities in the diabetic vascular wall. Oil Red O staining showed prominent lipid accumulation in diabetic vessels, which was markedly reduced following PTX administration (Fig. 3B). Hematoxylin–eosin staining demonstrated medial thickening and disorganized vascular architecture in diabetic mice, whereas PTX improved structural integrity (Fig. 3C). Immunofluorescence staining showed a marked reduction in GPX4 signal in diabetic aortas, and PTX restored GPX4 fluorescence intensity (Fig. 3D). Together, these observations confirm that PTX modulates the KLF10/GPX4 axis *in vivo* and improves ferroptosis-associated vascular alterations in diabetes.

## Discussion

In this study, we demonstrate that high glucose induces ferroptotic injury in vascular smooth muscle cells, as evidenced by increased KLF10 expression, suppression of GPX4, lipid peroxidation, and iron accumulation. Pentoxifylline (PTX) mitigated these abnormalities in a dose-dependent manner and alleviated vascular remodeling in diabetic mice, supporting a central role for the KLF10/GPX4 regulatory axis in hyperglycemia-induced ferroptosis. To our knowledge, this is the first study to directly link KLF10 to GPX4 repression and ferroptotic vulnerability in vascular smooth muscle cells under hyperglycemic stress. Moreover, while PTX is recognized to confer general vasculoprotective and antioxidant benefits, our work identifies a distinct and novel mechanism whereby PTX suppresses the KLF10/GPX4 axis to restrain ferroptosis. These findings provide mechanistic insights into how transcriptional dysregulation contributes to ferroptotic vulnerability in diabetic vasculature.

Although ferroptosis has been implicated in diabetic nephropathy, retinopathy, endothelial dysfunction, and cardiomyopathy (18), its relevance to vascular smooth muscle biology remains insufficiently explored. Our results align with established evidence that impaired GPX4 activity sensitizes cells to iron-dependent lipid peroxidation. Prior vascular-protective interventions – including structured exercise (19), metformin (20), and Nrf2 pathway activation (21) – primarily enhance antioxidant defenses. In contrast, PTX appears to exert a more upstream effect by inhibiting hyperglycemia-induced KLF10 upregulation, thereby restoring the transcriptional capacity of GPX4. How PTX downregulates KLF10 is not fully defined. Notably, KLF10 was originally identified as a TGF-β-inducible early gene (TIEG1), suggesting that attenuation of TGF-β-driven transcriptional programs represents a plausible upstream route to reduced KLF10 expression. Consistent with this possibility, PTX has been reported to suppress TGF-β1 expression and/or TGF-β/Smad2/Smad3 signaling in experimental settings and to blunt TGF-β1-associated pro-fibrotic or EMT-related responses, often in parallel with reduced inflammatory signaling (22). In addition, PTX has been shown to engage AMPK-related signaling in several models (23), and AMPK activation can antagonize canonical TGF-β/Smad signaling output (e.g., by limiting Smad2/Smad3 activation or downstream transcriptional activity) (24). Collectively, under hyperglycemic conditions, PTX may indirectly downregulate KLF10 by dampening upstream stress-responsive pathways – such as TGF-β/Smad-linked pro-fibrotic programs and/or inflammatory signaling – with AMPK acting as a potential modulatory node. Importantly, these mechanisms remain speculative in the absence of direct pathway interrogation; future studies using pathway-focused inhibition/activation, genetic perturbation, and promoter/reporter assays will be required to delineate the causal signaling cascade linking PTX exposure to KLF10 regulation. This distinction positions PTX as a mechanistically complementary approach within the expanding repertoire of ferroptosis-targeted strategies for diabetic vasculopathy.

Clinical management of diabetic vascular injury typically requires comprehensive control of glycemia, lipids, blood pressure, and microcirculatory function (25). PTX is widely used to improve hemorheology and endothelial perfusion (26). In the present work, however, we intentionally evaluated the direct, isolated molecular actions of PTX under hyperglycemic conditions, independent of other clinical variables. This reductionist approach allowed us to dissect the specific interaction between PTX and the KLF10/GPX4 axis, providing mechanistic clarification that complements rather than replaces established multifactorial therapies.

Members of the Krüppel-like factor family regulate redox signaling and lipid metabolism in diverse tissues. Whereas KLF2 and KLF4 exert vasoprotective antioxidant functions (27), KLF10 participates in stress-responsive transcriptional programs and has recently been linked to ferroptosis regulation (28). Similarly, GPX4 is a central regulator of ferroptosis in multiple organs, and disruption of GPX4-dependent lipid detoxification contributes to diseases such as sepsis, neurological disorders, ischemia–reperfusion injury, cardiovascular disease, and cancer (29). PTX has been reported to mitigate oxidative and iron-related injury in hepatic inflammation, renal ischemia–reperfusion, and neuroinflammation (30), although its relevance to ferroptosis has remained unclear. By pinpointing the KLF10/GPX4 axis as a PTX-responsive pathway, our findings refine the understanding of PTX action from a broadly ‘antioxidant’ effect to a defined transcriptional mechanism that limits ferroptotic stress in vascular smooth muscle cells.

Despite the protective effects observed, PTX only partially restored ferroptosis-related parameters, suggesting that upstream transcriptional modulation reduces susceptibility but does not fully reverse ferroptotic progression once established. These observations support the potential value of combination strategies – for example, PTX alongside direct ferroptosis inhibitors such as liproxstatin-1 or ferrostatin-1 – which may yield synergistic vascular protection in diabetes (31).

Several limitations should be acknowledged. First, the *in vivo* experiments were performed only in male mice. Although this design reduces variability, it also limits the extent to which the present findings can be generalized, as sex-related factors (including hormonal influences and potential differences in iron metabolism, redox homeostasis, and lipid peroxidation) may modulate ferroptosis susceptibility and vascular remodeling. Second, the sample size was chosen based on preliminary variability estimates and may not be powered to detect smaller structural changes. Future studies with larger cohorts, and importantly with inclusion of both male and female mice, will be needed to determine whether the KLF10/GPX4 axis and the protective effects of PTX are similarly observed across sexes and to clarify any sex-dependent differences. Although our results support a KLF10-dependent mechanism, combined KLF10 knockdown and PTX treatment was not performed; given that KLF10 depletion alone strongly restores GPX4, additional PTX effects would be challenging to interpret mechanistically. Moreover, other ferroptosis-related pathways (including system Xc^−^, FSP1–CoQ10 (32), and mitochondrial lipid remodeling (33)) were not examined and may interact with the KLF10/GPX4 axis.

In conclusion, this study identifies the KLF10/GPX4 pathway as a key mediator of ferroptosis in diabetic vascular smooth muscle cells and demonstrates that PTX partially attenuates ferroptotic stress both *in vitro* and *in vivo*. These findings deepen the understanding of ferroptosis regulation in diabetic vascular disease and highlight PTX – potentially in combination with direct ferroptosis inhibitors – as a promising therapeutic strategy for mitigating diabetic vascular complications.

## Conclusions

This study demonstrates that high glucose induces ferroptosis in vascular smooth muscle cells through KLF10-mediated repression of GPX4, leading to iron accumulation, lipid peroxidation, and excessive reactive oxygen species generation. PTX attenuates these effects by suppressing KLF10 expression and restoring GPX4 activity, thereby reducing ferroptotic stress *in vitro* and mitigating vascular remodeling in diabetic mice. These findings highlight the KLF10/GPX4 axis as a mechanistic contributor to diabetic vascular injury and support the potential of PTX as a therapeutic candidate for ferroptosis-associated vasculopathy. Future studies, including sex-inclusive and clinically translational investigations, will be essential to further validate the efficacy and therapeutic scope of ferroptosis-targeted interventions.

## Supplementary materials



## Declaration of interest

The authors declare that there is no conflict of interest that could be perceived as prejudicing the impartiality of the work reported.

## Funding

This work was supported by Sichuan Western Spirit–Shiyao Pharmaceutical Co., Ltd (Grant No. 2HC2021046); the Second Hospital of Hebei Medical University (Grant No. 2HN202106); the Hebei Provincial Department of Finance (2026 Government-funded Clinical Medicine Outstanding Talent Training Program; Grant No. ZF2026136); and the Open Research Fund of Key Laboratory of Endocrine Glucose & Lipids Metabolism and Brain Aging, Ministry of Education (Open Research Fund No. 2024JYBZDSYS012).

## Data availability

The datasets used or analyzed during the current study are available from the corresponding author on reasonable request.
