# Supplementary material for: Protective effect of pentoxifylline against high-glucose-induced ferroptosis in vascular smooth muscle cells
Source: J Mol Endocrinol. 2026 Apr 24;76(3):e250086. doi: 10.1530/JME-25-0086 (PMC13130825; doi:10.1530/JME-25-0086)

Supplementary Figure S1. Metabolic characterization of the in vivo cohorts during the 24-week study. (A) Body weight and (B) random (non-fasting) blood glucose were monitored at predefined time points in WT, db/db, WT+PTX, and db/db+PTX groups. db/db mice were fed a high-fat diet, and PTX treatment was initiated at week 16 and continued for 8 weeks (week 16–24). Data are presented as mean ± SEM; n = 6 per group

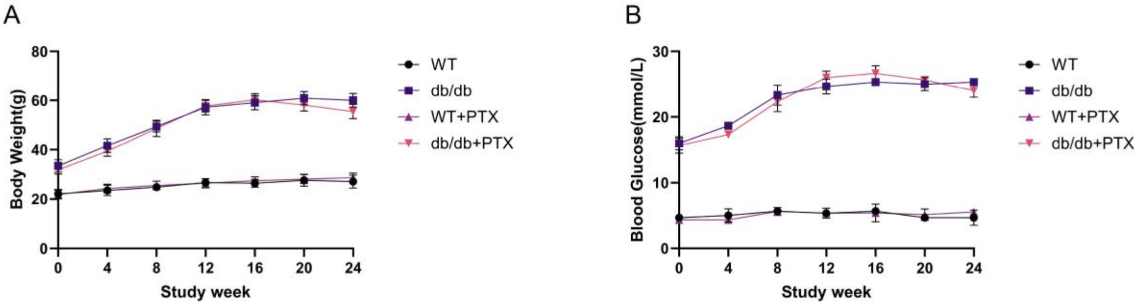

Supplement: Supplementary file 1 [file supplementary_materials.pdf]
